# Laryngeal Lymphoid Hyperplasia Presenting As Stridor in Pediatric Age

**DOI:** 10.22038/IJORL.2021.58575.3024

**Published:** 2022-03

**Authors:** Dillip-kumar Samal, Anindya Nayak, Amit-Kumar Adya

**Affiliations:** 1 *Deparment of ENT & Head and Neck Surgery, All India Institute of Medical Scieneces*, *Bhubaneswar, India.*; 2 *Deparment of Pathology & Lab Medicine, AIIMS, Bhubaneswar, India.*

**Keywords:** Glottis, Subglottis, Tracheostomy, Stenosis, Stridor, Lymphoid hyperplasia

## Abstract

**Introduction:**

Benign lymphoid hyperplasia uncommonly involves the larynx. Involvement of glottis and subglottis is even rare, considering sparse lymphatic supply compared to supraglottis.

**Case Report:**

A young female presented to emergency with worsening breathing difficulty. After securing the airway, she had found to have circumferential glottis and subglottic mucosa covered firm swelling. Histopathological evaluation of the swelling showed it to be benign lymphoid hyperplasia. Coblation assisted excision of the lesion was done, and the patient became asymptomatic without any recurrence.

**Conclusion:**

Idiopathic lymphoid hyperplasia is a very rare entity to present as glottis and subglottic lesions. Probably, it's the first case to be reported in the literature as laryngeal involvement sparing the supraglottis.

## Introduction

Benign lymphoid hyperplasia or ectopically placed tonsillar tissue is not rare in sites other than waldeyer’s ring. It usually presents as lymphoid aggregate and may be found in ventral surface of the tongue, floor of the mouth, soft palate, and even in the larynx (1). In the larynx, the various subsites involved are arytenoids, aryepiglottic folds, false vocal cords and vocal cords as per literature reviewed. Laryngeal involvement presenting as airway stenosis has never been described in the literature and more so in the pediatric age group. Unlike adults, in children it may present early with breathing difficulty, as in our case. We had a young female with glottic and subglottic airway stenosis presented in emergency with breathing difficulty and stridor, and managed successfully by surgical excision of the mass. 

## Case Report

**Fig 1:**
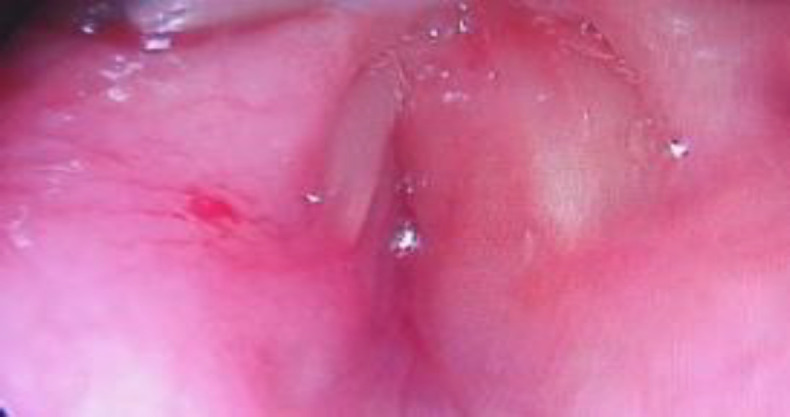
Direct laryngeal endoscopic picture at presentation showing almost complete glottic and subglottic stenosis

A 14-year-old female presented with progressively worsening difficulty in breathing over five months. She had associated change in voice for the same duration. The patient had a foreign body sensation in her throat with mild odynophagia. She did not have any cough during oral intake. She did not have any associated co-morbidities or any relevant family history. On initial evaluation with a chest and neck skiagram, significant upper airway narrowing was found. Due to the worsening stridor, the patient underwent an emergency tracheostomy. Direct laryngoscopy and evaluation was also done in the same setting under general anaesthesia. The findings revealed a circumferential smooth mucosa-covered bulge arising from the under-surface of bilateral true vocal cords extending up to 15 mm below its level, causing a near-complete subglottic obstruction (Figure 1). 

Biopsy was taken from the subglottic mass and sent for histopathological examination. The supraglottic larynx was normal. Vocal cords mobility couldn't be assessed because of the near-complete stenosis. Contrast-enhanced computer tomography Neck and thorax was done, which showed an asymmetrical circumferential wall thickening with mild enhancement involving the subglottic larynx causing severe airway compromise. The histopathological evaluation of the mass reported lymphoid hyperplasia. The patient was then evaluated further to rule out lymphoma or other lymphoproliferative disorders. 

Any granulomatous condition or auto-immune disorders were ruled out. The nasal endoscopic evaluation showed adenoid hyperplasia, and the rest of the systemic examination was normal. 

The patient was given a course of oral and inhalation steroid therapy for six weeks. She responded well initially, with subsequent laryngeal endoscopies showing a 30-40% reduction in the size of the mass. Keeping in mind the patient's young age, a decision to taper and eventually stop the steroid therapy was taken. 

**Fig 2:**
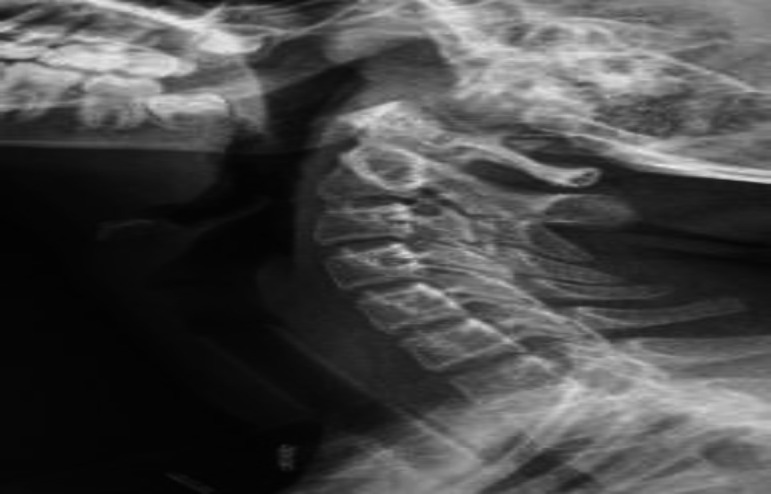
Soft tissue neck X-Ray showing Montgomery T-tube placed as a stent following Coblation release of stenosis

However, over the next few weeks, the mass was found to again increase in size. As medical treatment didn't help, surgical intervention was planned. Coblation assisted removal of the glottis-subglottic lesion was done circumferentially preserving bilateral vocal cords. A Montgomery T- tube was placed as a stent to maintain the subglottic lumen and to prevent restenosis (Figure 2). 

**Fig 3:**
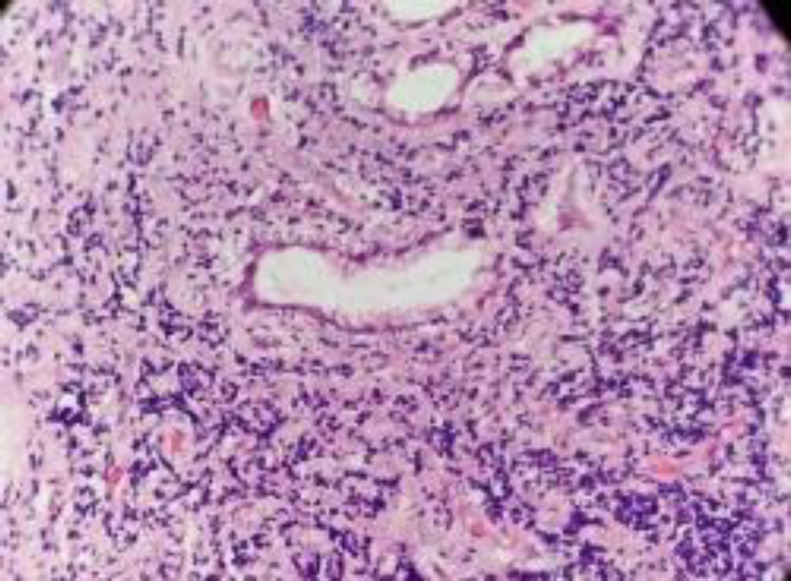
H &E stain, 400 x showing lymphoid infiltration in the subepithelium with entrapment of mucosal glands

The excised mass was again analyzed histopathologically by two independent pathologists. The microscopic analysis shows fragments of mucosa lined by squamous epithelium, and sub-epithelium shows multiple foci of lymphoid infiltration with entrapment of mucosal glands (Figure 3). 

The infiltrate was composed of both lymphocytes and plasma cells. There was no evidence of granuloma or malignancy seen. Thus, a diagnosis of ‘Idiopathic lymphoid hyperplasia’ was made. 

**Fig 4:**
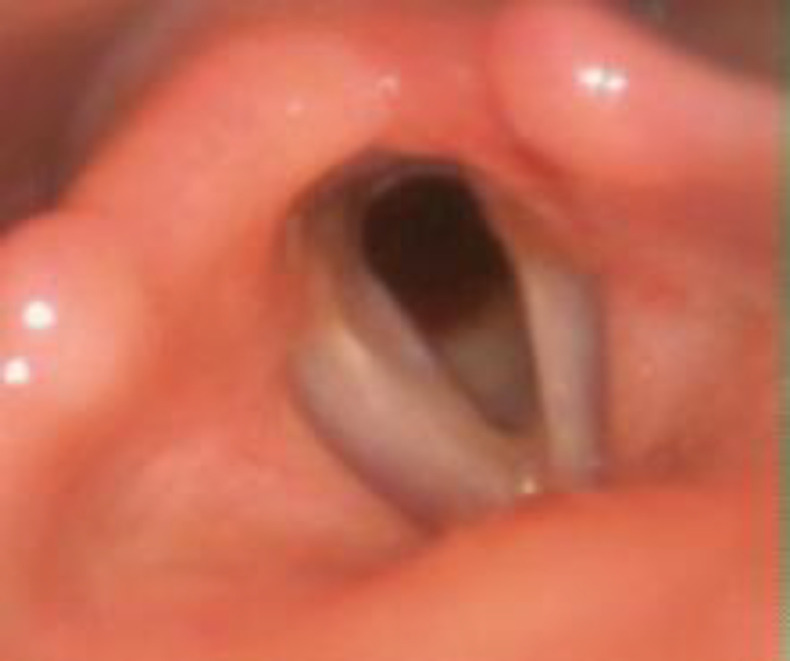
Laryngoscopic picture on follow-up showing adequate glottic lumen

Twelve weeks later, she was evaluated, and the glottic and subglottic airway seems normal on X-ray. Montgomery tube was removed, and tracheostomy tube was placed, and corking was done. On follow-up, the glottic and subglottic lumen were found adequate, and she was decannulated (Figure 4). She is asymptomatic at one year follow-up without any disease recurrence. 

## Discussion

The larynx has a huge lymphatic supply, especially the supraglottic part. Thus, various lymphatic disorders also can involve the larynx either primarily or a part of systemic involvement. Primary lymphatic disorders of the larynx are very uncommon, which include mucosa-associated lymphoid tissue lymphoma (MALT-type lymphoma), a form of Non-Hodgkin lymphoma, or maybe a presentation of true lymphoma. The presence of benign focal nodular hyperplasia in the larynx has been reported infrequently in the literature. It may present with some foreign body sensation, change in the voice, or breathing difficulty. In our case, focal lymphoid hyperplasia involved the glottis and subglottis, which is very rare considering very sparse lymphatic circulation. Also, presentation at 14-year age is very uncommon for this age. Regarding laryngeal lymphoid hyperplasia, probably the first case was reported by Climie ARW, Waggoner LG, and Krabbenhoft KL in 1964 as lymphoid hamartoma of the larynx (2). The case was treated successfully by surgical excision of the mass. A rare case of lymphoid hyperplasia of the adult larynx (hyperplasia of the laryngeal tonsil) was reported by Pellettiere E V, Holinger LD, Schild JA in 1980 (3). While this benign lesion recurred following surgical excision, the patient was followed for more than two years without additional therapy and was asymptomatic. Kiminori Sato et al., in 1991, reported a case of 51 years old female presented with left false vocal cord lymphoid hyperplasia. It was treated by Suprahyoid pharyngotomy and enucleation of the lesion (4). The patient had no evidence of any recurrence in another two and half years of follow-up. Bandino F, Kenway B et al. in 2020, reported a case of ectopic laryngeal tonsil in a 66 years' female who presented with dysphonia. She was treated by micro laryngeal surgery and excision of the lesion from the left vocal process of the arytenoid (5). Histopathology was suggestive of benign lymphoid nodule with reactive follicles and epithelial crypts lined by stratified squamous epithelium. In many instances, lymphoid hyperplasia confuses low-grade lymphoma like mucosa-associated lymphoid tissue lymphoma (MALT-type lymphoma). Few authors usually use the term pseudolymphoma for that, which had been treated as malignancy (6). This MALT-type lymphoma shows proliferation of lymphoplasmacytic cells and the presence of lymphoepithelial lesions and germinal centers (7). Whereas in our case, the epithelium was lined by squamous epithelium, and the sub-epithelium was showing the presence of lymphoid aggregates. Idiopathic lymphoid hyperplasia, unlike MALToma, is a purely benign condition, where surgery as monotherapy is adequate. 

## Conclusion

Airway obstruction is an emergency and more often needs urgent intervention and expertise, especially in pediatric age group. The various causes in the young populations are inflammatory, auto-immune, traumatic, or maybe neoplastic. Idiopathic lymphoid hyperplasia is a rare cause of airway stenosis, which needs to be addressed by surgical excision. It should be differentiated from other lymphoid disorders like Lymphoma or MALT-type lymphoma. With Strong clinical suspicion and proper histopathological evaluation only, we can reach the diagnosis. 
